# An Epidemic of Purulent Inflammation of the Milk-ducts, Affecting Seventy Cows1From the Bacteriological Laboratory of the Health Department of Baltimore.

**Published:** 1897-03

**Authors:** William Royal Stokes, A. W. Clement

**Affiliations:** Bacteriologist to the Health Department and Lecturer on Bacteriology to the Baltimore Medical College, Baltimore; State Veterinarian


### AN EPIDEMIC OF PURULENT INFLAMMATION OF THE MILK-DUCTS, AFFECTING SEVENTY COWS.[1]

By William Royal Stokes, M.D.,
BACTERIOLOGIST TO THE HEALTH DEPARTMENT AND LECTURER ON BACTERIOLOGY TO THE BALTIMORE MEDICAL COLLEGE, BALTIMORE;

AND

A. W. Clement, D.V.S.,
STATE VETERINARIAN.

The first clinical observation of the probable transmission of disease through milk dates back as far as 1764, when Sagar described a number of throat affections and aphthous ulcers of the mouth which he attributed to drinking the milk from a certain cow. This communication seems to have attracted but little attention at the time, but its correctness has of late been confirmed by the objective demonstration of various pathogenic bacteria in the milk of cows, especially in that of animals suffering from an inflammatory condition of the udder and teats, called garget.

Nivens has described an epidemic of diarrhœa affecting 160 persons in which the source of infection was traced to the milk from a cow affected with the above disease. The cultures from this milk showed the presence of the bacillus coli communis and the streptococcus pyogenes. Kruger, in 1890, was one of the first to find the pyogenic cocci in milk of diseased cows. He observed many pus-

[1] From the Bacteriological Laboratory of the Health Department of Baltimore.

cells in a case of suspected bovine tuberculosis, but failed to demonstrate the presence of the bacillus tuberculosis. He observed, however, many groups of cocci, which he proved to be staphylococcus pyogenes aureus, capable of producing subcutaneous abscesses in rabbits. Guillebeau also examined the milk of seventy-six cows with inflammation of the udders, and found in all of the cases varieties of the pyogenic cocci, which were often virulent when inoculated into animals. Further, similar cases are reported by Karlinski, Escherich, Longard, and Adamitz.[1]

*Clinical Observations.* I was called upon professionally in August to attend a herd of cattle which, as the owners said, were "milking pus." I found a herd of about one hundred cows, all affected to a greater or less extent. They were all nearly dry, and what milk could be obtained was thick and yellowish. The cows stood in a double row of stanchions. The history, obtained after careful inquiry, was that the disease first appeared in one cow; that the owner's attention was called to the condition of the milk by the retailers who bought it. The infection spread to the rest of the herd with great rapidity, so that in the course of two or three weeks the whole herd had become infected.

These cattle were at the time on pasture, fed twice a day on mill-feed, and, according to the foreman's statement, milked regularly. Further inquiry brought forth the information that a strange man had hired out on the farm, who was an experienced milker, but who sought professional advice from the physician attending the family for a sore upon his finger, which he said he got from milking cows on a large dairy-farm in York, Pa. This man left the place in about a week, and in a few days after his departure the disease appeared in the first cow, soon followed by its appearance in the rest of the herd. Cleanliness and irrigation with warm water gradually caused the animals to become dry, in which condition they have remained up to the present time.

A complete autopsy was made upon one of the cows, but nothing abnormal was made out with the exception of a purulent inflammation of the somewhat dilated milk-ducts. Cultures from the blood of the heart and the internal viscera remained sterile.

*Histological Examination.* Sections through the diseased portions of the mammary gland show that the larger milk-ducts are dilated and are either empty or plugged up by masses of polynuclear leucocytes. This condition, however, is much more marked

[1] References from Report of the Health Officer of the District of Columbia for 1895.

in the secreting acini of the gland. These are often markedly dilated, and for the most part contain large or small accumulations of pus-cells. This process ends here, and there is no sign of inflammation in the surrounding connective tissue, the suppuration having limited itself to the milk-ducts and acini of the gland. Although we were only able to secure one autopsy, yet the absence of any local swelling in the mammary glands of the other cows would seem to show that the condition was a similar one in the rest of the herd. The lymphatic glands surrounding the mamma were found to be normal.

*Bacteriological Examination.* The cultures were obtained from two different cows. The methods of procedure were similar in both cases and the results identical. The teat was carefully washed with soap and water, and the creamy fluid was then squirted from an affected teat into a sterile tube without allowing the teat to come into contact with the opening of the test-tube. Cultures were made soon afterward at the laboratory.

Cover-slips showed that this fluid consisted entirely of pus containing a moderate number of short chains of streptococci. Plate-cultures in twenty-four hours showed a moderate number of fine, pin-point gray colonies which consisted of short chains of cocci, staining by Gram's method and the ordinary aniline dyes. The organisms grew invisibly on potato, coagulated and acidulated milk in twenty-four hours, formed small gray colonies on "slant" agar, turned litmus agar-red, and grew in fairly long chains in bouillon. Gelatin was not liquefied.

*Inoculations.* White mice were inoculated subcutaneously with the pus from the udders and with pure agar-cultures. Guinea-pigs were also inoculated subcutaneously with the pus, and were given one cubic centimetre of a pure fluid-culture of the organism. All of the animals survived these inoculations for several weeks. The capriciousness of the streptococcus as regards virulence, however, is well known, and this fact does not disprove its species.

From our experiments, we are of the opinion that we have demonstrated the presence of the streptococcus pyogenes, possessing a low grade of virulence.

The study of this epidemic presents several points of interest. Garget has been caused experimentally by wounding the udders, and allowing the wound to come into contact with various non-sterile materials. Assuming that one or several of the herd may have become affected in a similar fashion, or from contact with an infected finger, the gradual transferrence of the disease from cow to cow can be plainly traced to the hands of the milker, from the history of the epidemic. The similarity of the process to that of gonorrhœal inflammation of the mucous membrane in man is also a point of considerable interest, although, of course, the causes differ.

From our observations gained from the routine microscopical and bacteriological examination of milk, we believe that the dangers of infection can be materially lessened by the observance of a few simple precautions, and by the use of the microscope. Clean hands, milking utensils, udders, and stables are, of course, important.

We have also found many pus-cells in the centrifugalized sediment obtained from the mixed specimens from several large herds, and in one instance we were able to trace the source of the pus to one cow having a local abscess of the udder. After the isolation of this animal we were unable to demonstrate the presence of any more pus in the milk from the rest of the herd.

Although we are aware that it is impossible to obtain a perfectly sterile milk directly from the udder of a healthy cow, yet we believe that the presence of many colonies of the staphylococcus pyogenes aureus or streptococcus pyogenes, when obtained from a "slant" culture of a single platinum loop made from the milk drawn according to the method mentioned above, renders this fluid unfit for use, especially for infants.

Cultures thus made from the last portion of the milk drawn from a healthy cow are usually either sterile or contain only a few colonies of a white coccus or other bacteria. On the other hand, the milk of animals suffering from garget shows many colonies of the pus-cocci and often contain many pus-cells.

